# Prevalence and sociodemographic factors of depression, anxiety and stress in Saudi Arabia: a survey of respiratory therapists

**DOI:** 10.3389/fpsyt.2024.1289456

**Published:** 2024-02-20

**Authors:** Abdulelah M. Aldhahir

**Affiliations:** Respiratory Therapy Department, Faculty of Applied Medical Sciences, Jazan University, Jazan, Saudi Arabia

**Keywords:** depression, anxiety, stress, respiratory therapist, Saudi Arabia

## Abstract

**Background:**

Depression, anxiety and stress are prevalent among healthcare providers but limited data is available regarding respiratory therapists (RTs). This study aimed to assess the prevalence of depression, anxiety and stress, and identify the associated sociodemographic factors among RTs in Saudi Arabia.

**Methods:**

A cross-sectional online survey was distributed to RTs in Saudi Arabia. Data were summarized using frequency and percentages. Stress, anxiety, and depression prevalence rates were expressed as proportions with 95% confidence intervals (CI). The associated factors of stress, anxiety, and depression were subjected to logistic regression.

**Results:**

Overall, 988 (91%) RTs, 661 (66.9%) males, completed the online survey. The prevalence (95% CI) of depression, anxiety and stress among RTs was 81.3% (0.787, 0.837), 89.6% (0.875, 0.914), and 45.5% (0.424, 0.487), respectively. RT staff who were ≥41 years old, divorced, widowed or separated, or had > 10 years of clinical experience were more likely to experience stress. RTs who were (divorced, widowed or separated), did not live with their family, were current smokers, or worked the night shift were more likely to experience anxiety. RTs who were (divorced, widowed or separated), were current smokers, had >10 years of clinical experience were more likely to experience depression.

**Conclusion:**

Stress, anxiety and depression are prevalent among RTs. Several sociodemographic factors are associated with the incidence of stress, anxiety and depression.

## 1 Introduction

Depression is a mental health condition characterized by persistent feelings of melancholy, despair, and boredom regarding activities (1). It can substantially influence a person’s mental well-being and everyday functioning. Work-related stress, a heavy workload, occupational burnout, and a lack of social support are possible causes of depression among healthcare providers (2, 3). Several national and international studies have reported the prevalence of depression among healthcare workers ranging from 18% to 36% (4–7). Meanwhile, the reported prevalence of depression among healthcare workers in Saudi Arabia was between 24% and 80% (8–10). Among RTs, the reported prevalence was 58% (11). According to D’Alessandro-Lowe et al., in a study conducted in 2023 among Canadian RTs, symptoms of depression were reported in 52% of the total Canadian RTs who participated in the study (12). Depression may have negative implications on RTs, ranging from lower job satisfaction and higher absenteeism to poor patient care quality (13). Tackling this crucial issue is important for both RTs and patients.

Anxiety is a mental health condition characterized by excessive worry and uneasiness. It has a significant impact on an individual’s mental state, emotions, and physical well-being. Anxiety can be caused by various circumstances, including job-related stress, difficult patient situations, a lack of control over the work environment, and a fear of making mistakes (14). These variables may lead to increased anxiety among these healthcare practitioners. In addition, anxiety may harm RTs’ well-being, work performance, and interpersonal connections with colleagues and patients. Several studies have investigated and reported the prevalence of anxiety among healthcare workers ranging between 21% and 51% (12, 15, 16), while the reported prevalence of anxiety among healthcare workers in Saudi Arabia was between 24% and 56% (8, 10, 17, 18). Among RTs, the reported prevalence of anxiety was 21% (16). Thus, understanding and treating anxiety is critical for increasing RTs’ overall performance.

Stress is a physiological and psychological reaction to difficult circumstances. It can come from long working hours, difficult patient situations, ethical quandaries, and exposure to traumatic events. It can influence an individual’s overall well-being (19). Stress may harm RTs’ physical and mental health, as well as their work satisfaction and the quality of patient care they deliver (20). Researchers have reported that stress is prevalent among healthcare providers with a rate of 9% to 18% (21, 22), while the reported prevalence of stress among RTs was around 51% (12).

Several sociodemographic factors such as age, gender, marital and smoking status, night shift duty, and clinical experience have been reported in the literature that contributed to the development of mental health conditions among healthcare workers (23–29).

In Saudi Arabia, RT is an essential healthcare profession that treated patients with all ages who suffer from respiratory related health problem in critical, non-critical, emergency, and non-emergency healthcare settings. RTs mainly work in intensive care units and emergency departments, which are very stressful environments, may experience mental health conditions due to high workload, staff shortages, and long hour shifts.

There is limited data on the prevalence of depression, anxiety and stress among RTs in Saudi Arabia, especially following the COVID-19 pandemic. Therefore, this study aimed to assess the prevalence of depression, anxiety and stress, and identify the associated sociodemographic factors of depression, anxiety and stress among RTs in Saudi Arabia following the COVID-19 pandemic. We hypothesized that depression, anxiety and stress are prevalent among RTs staff in Saudi Arabia and several sociodemographic factors may contribute the development of theses mental health conditions.

## 2 Methods

### 2.1 Study design

A cross-sectional online survey was conducted and distributed between the 14^th^ of March and 30^th^ of June 2023 using the Survey Monkey platform.

### 2.2 Instrument

The questionnaire consisted of two sections as follows. Section one comprised nine questions regarding demographical information such as age, gender, years of clinical experience, marital and smoking status, living arrangements, and academic qualifications. Section two comprised the depression, anxiety and stress scale-21 (DASS-21), which is a popular self-reporting tool for assessing the extent of depression, anxiety, and stress symptoms, with established validity and reliability among the general papulation of Saudi Arabia; Cronbach alpha value equal to 0.94 and an internal reliability coefficient for the depression (0.88), anxiety (0.81), and stress (0.89) subscales (30–32). DASS-21 consists of 21item: seven items for depression, seven items for anxiety and seven items for stress. The items use a four-point Likert scale where zero is “did not apply to me at all”, one is “applied to me to some degree”, two is “applied to me a considerable degree”, and three is “applied to me very much”.

For the depression subscale interpretation, a score between zero and nine is considered normal, a score between ten and 13 is considered mild, a score between 14 and 20 is considered moderate, a score between 21 and 27 is considered severe, and a score of ≥ 28 is considered to reflect extremely severe depression. For the anxiety subscale interpretation, a score between zero and seven is considered normal, a score between eight and nine is considered mild, a score between ten and 14 is considered moderate, a score between 15 and 19 is considered severe, and a score of ≥ 20 is considered extremely severe. For the stress subscale interpretation, a score between zero and 14 is considered normal, a score between 15 and 18 is considered mild, a score between 19 and 25 is considered moderate, a score between 26 and 33 is considered severe, and a score of ≥ 34 is considered extremely severe.

The objectives of the study, information on the chief investigator, and the time required to complete the survey were provided in the cover letter of invitation. No personal data were collected; all the participants consented to participation and voluntary participation was confirmed by asking potential participants if they were happy to be in the study.

### 2.3 Data collection and sampling strategy

Convenience sampling strategy was utilized to recruit potential participants. Respiratory therapists who work in Saudi Arabia were the main target of the study and were invited via Professional committee such as Saudi Society of Respiratory Care and social networks (Twitter, WhatsApp, and Telegram).

### 2.4 Sample size

The target sample size was estimated based on the WHO recommendations for the minimal sample size needed for a prevalence study (33). Using a confidence interval of 95%, a standard deviation of 0.5, a margin of error of 5%, the required sample size was 385 participants.

### 2.5 Ethical approval

Institutional Review Board approval for the study was sought from King Abdulaziz University with reference number (EC2023-026).

### 2.6 Statistical analysis

The Statistical Package for Social Sciences (SPSS software, Version 27) was used to analyze the data. Frequency and percentages were used to summarize demographic characteristics. stress, anxiety, and depression prevalence rates were expressed as proportions with 95% confidence intervals (CI). The associated factors of stress, anxiety, and depression, such as age, gender, marital status, living arrangement, academic qualification, hospital sector, years of experience, working shift, and smoking status, were subjected to a univariate binary logistic regression analysis. We divided our participants into two different groups for each subscale (depression, anxiety, and stress) based on DASS-21 interpretation. The associated sociodemographic factors of depression between group one who scored between zero and nine (normal), and group two who scored more than 9 were subjected to a univariate binary logistic regression analysis. The associated sociodemographic factors of anxiety between group one who scored between zero and seven (normal) and group two who scored more than seven were subjected to a univariate binary logistic regression analysis. The associated sociodemographic factors of stress between group one who scored between zero and 14 (normal) and group two who scored more than 14 were subjected to a univariate binary logistic regression analysis. Multivariate regression analysis was applied to examine the adjusted odds ratio for associated factors that was identified significant in the bivariate logistic regression. A P value of < 0.05 was considered a statistically significant.

## 3 Results

### 3.1 Sociodemographic characteristics

Overall, a total of 1,082 RTs responded to the online survey. Of these, 94 respondents were excluded due to missing more than one entry in the DASS-21 questionnaire. Ultimately, 988 (91%) RTs, of which 661 (66.9%) were males, were included. The majority 775 (78.4%) were between 20 to 30 years old. Around 29% were from the Western region, followed by 27% from the Central region and 22% from the Eastern region. More than half of the respondents reported being single 559 (60.6%), non-smokers 665 (66.30%), and living with their family 670 (80.70%). A high proportion of the respondents held a bachelor’s degree (780: 78.9%) and 339 (40.4%) had between one and five years of clinical experiences. The full sociodemographic characteristics are summarized in **Table 1**.

**Table 1:** Demographic data of study respondents (n= 988).

Demographics	Frequency (%)
Gender	
Male Female	661 (66.9%) 327 (33.1%)
Age	
20 – 30 31 – 40 > 41	775 (78.4%) 162 (16.4%) 213 (5.1%)
Geographic Location	
Central Region Western Region Eastern Region Southern Region Northern Region	267 (27%) 286 (29%) 217 (22%) 168 (17%) 50 (5%)
Marital Status	
Single Married Divorced, widow, or separated	599 (60.6%) 300 (30.4%) 89 (9%)
Smoking Status	
Non-smoker Former smoker Current smoker	655 (66.3%) 172 (17.4%) 161 (16.3%)
Living arrangement	
Living with family Not living with family	670 (67.8%) 318 (32.2%)
Academic qualification	
Associate degree Bachelor degree Post-graduate degree	121 (12.2%) 780 (78.9%) 87 (8.8%)
Years of clinical experience	
< 1 1 – 5 6 – 10 > 10	338 (34.2%) 339 (40.4%) 154 (15.6%) 97 (9.7%)
Hospital sector	
Governmental Private	797 (80.7%) 191 (19.3%)
Working shift	
Day Night	707 (71.6%) 281 (28.4%)

Data are presented as frequency and percentage.

**Table 2:** The severity of stress, anxiety and depression among respondents (n= 988).

	Severity	Frequency (%)
**Stress**	Normal	538 (54.50%)
	Mild	296 (30%)
	Moderate	141 (14.30%)
	Severe	13 (1.3%)
	Extremely Severe	0
**Anxiety**	Normal	103 (10.40%)
	Mild	101 (10.20%)
	Moderate	369 (37.30%)
	Severe	306 (31%)
	Extremely Severe	109 (11%)
**Depression**	Normal	185 (18.70%)
	Mild	263 (26.60%)
	Moderate	458 (46.40%)
	Severe	73 (7.40%)
	Extremely Severe	9 (0.90%)

Data are presented as frequency and percentage.

### 3.2 Prevalence of stress, anxiety and depression among RT staff

The prevalence (95% CI) of stress among RTs was 45.5% (0.424, 0.487). The mean (±SD) of the DASS-21 scores for the stress subscale was 14 (±0.14), indicating a normal level of stress among RTs. The prevalence (95% CI) of anxiety was 89.6% (0.875, 0.914). The mean (±SD) of the DASS-21 scores for the anxiety subscale were 13.75 (±0.14), indicating a moderate level of anxiety among RTs. The prevalence (95% CI) of depression among the respondents was 81.3% (0.787, 0.837). The mean (±SD) of the DASS-21 scores for the depression subscale was 14 (±0.14), indicating a moderate level of depression among RTs. The full details about the prevalence and severity of stress, anxiety, and depression are presented in **Table 2**.

### 3.3 Associated sociodemographic factors of stress among RTs’ staff

Regarding stress, the binary logistic regression analysis revealed that female RTs were one time more likely to experience stress compared to males [OR: 1.2 (95% CI: 1.2 to 1.79); p= 0.021]. RTs who were 41 years old and above were almost two times more likely to experience stress compared to those who were 20 to 30 years old [OR: 1.8 (95% CI: 1.03 to 3.27); p= 0.040]. Those who were (divorced, widowed or separated) were almost three times more likely to experience stress compared to those who were single [OR: 2.78 (95% CI: 1.73 to 4.48); p < 0.001]. Former smokers and current smokers were one time and two times more likely to experience stress compared to non-smokers RT staff [OR: 1.62 (95% CI: 1.16 to 2.27); p = 0.005] and [OR: 1.90 (95% CI: 1.32 to 2.65); p < 0.001]. RTs who do not live with their family were two times more likely to experience stress compared to those who lived with their family [OR: 2.11 (95% CI: 1.27 to 3.52); p = 0.004]. RTs with more than 10 years of clinical experience were almost two times more likely to experience stress compared to those with less than a year [OR: 1.81 (95% CI: 1.14 to 2.88); p = 0.012]. RTs who worked in private hospitals were one time more likely to experience stress compared to those who worked in government hospitals [OR: 1.44 (95% CI: 1.05 to 1.98); p = 0.024]. **Table 3** summarizes all the predictors of stress.

**Table 3:** Bivariate logistic regression models of the factors associated with stress, anxiety and depression.

*Stress subscale*	OR (95% CI)
Gender	
Male	1
Female	1.2 (1.05 to 1.79) p 0.021
Age	
20 – 30	1
> 41	1.83 (1.03 to 3.27) p 0.040
Marital Status	
Single	1
Divorced, widow, or separated	2.78 (1.73 to 4.48) p 0.000
Smoking Status	
Non smoke	1
Former smoker	1.62 (1.16 to 2.27) p 0.005
Current smoker	1.90 (1.32 to 2.65) p 0.000
Living arrangement	
Living with family	1
Not living with family	2.11 (1.27 to 3.52) p 0.004
Years of clinical experience	
< 1	1
> 10	1.81 (1.14 to 2.88) p 0.012
Hospital sector	
Governmental	1
Private	1.44 (1.05 to 1.98) p 0.024
*Anxiety subscale*	
Marital Status	
Single	1
Divorced, widow, or separated	4.61 (1.42 to 14.92) p 0.011
Living Arrangement	
Living with family	1
Not living with family	5.70 (1.32 to 24.44) p 0.019
Smoking Status	
Non-smoker	1
Former smoker	3.30 (1.57 to 6.96) p 0.002
Current smoker	6.33 (2.29 to 17.50) p 0.000
Hospital Sector:	
Governmental	1
Private	2.40 (1.22 to 4.68) p 0.011
Working Shift	
Day	1
Night	2.47 (1.38 to 4.42) p 0.002
*Depression*	
Gender	
Male	1
Female	1.47 (1.03 to 2.10) p 0.035
Marital Status	
Single	1
Married	2 (1.36 to 2.93) p 0.000
Divorced, widow, or separated	6.54 (2.35 to 18.15) p 0.000
Smoking Status	
Non-smoker	1
Former smoker	3.1 (1.78 to 5.30) p 0.000
Current smoker	3.91 (2.12 to 7.24) p 0.000
Living arrangement	
Living with family	1
Not living with family	2.28 (1.06 to 4.86) p 0.033
Years of clinical experience	
< 1	1
> 10	2.78 (1.41 to 5.45) p 0.003
Hospital sector	
Governmental	1
Private	1.76 (1.11 to 2.79) p 0.016
Working shift	
Day	1
Night	1.58 (1.06 to 2.35) p 0.023

CI, confidence interval; OR, odds ratio.

Multivariate regression analysis identified that RTs who were (divorced, widowed or separated) were almost three times more likely to experience stress compared to single RTs [AOR: 2.72 (95% CI: 1.65 to 4.47); p= 0.000]. Former smokers and current smokers were two times more likely to experience stress compared to non-smokers RT [AOR: 1.76 (95% CI: 1.24 to 2.49); p= 0.002] and [AOR: 2.04 (95% CI: 1.43 to 2.92); p = 0.000]. RTs who did not live with their family were two times more likely to experience stress compared to those who lived with their family [AOR: 2.10 (95% CI: 1.26 to 3.51); p = 0.004]. RTs with more than 10 years of clinical experience were almost two times more likely to experience stress compared to those with less than a year of experience [AOR: 1.86 (95% CI: 1.19 to 2.93); p = 0.007]. **Table 4** summarizes all the predictors of stress.

**Table 4:** Multivariate logistic regression models of the factors associated with stress, anxiety and depression.

*Stress subscale*	AOR* (95% CI)
Marital Status	
Single	1
Divorced, widow, or separated	2.72 (1.65-4.47) p 0.000
Smoking Status	
Non smoke	1
Former smoker	1.76 (1.24-2.49) p 0.002
Current smoker	2.04 (1.43-2.92) p 0.000
Living arrangement	
Living with family	1
Not living with family	2.10 (1.26-3.51) p 0.004
Years of clinical experience	
< 1	1
> 10	1.86 (1.19-2.93) p 0.007
Hospital sector	
Governmental	1
Private	1.41 (1.03-1.94) p 0.033
*Anxiety subscale*	
Marital Status	
Single	1
Divorced, widow, or separated	4.47 (1.34-14.94) p 0.015
Living Arrangement	
Living with family	1
Not living with family	5.48 (1.28-23.53) p 0.022
Smoking Status	
Non-smoker	1
Former smoker	3.36 (1.58-7.15) p 0.002
Current smoker	6.65 (2.39-18.48) p 0.000
Hospital Sector:	
Governmental	1
Private	2.32 (1.18-4.55) p 0.015
Working Shift	
Day	1
Night	2.31 (1.28-4.15) p 0.005
Depression	
Marital Status	
Single	1
Married	1.83 (1.20-2.79) p 0.005
Divorced, widow, or separated	5.91 (2.09-16.70) p 0.000
Smoking Status	
Non-smoker	1
Former smoker	3.15 (1.81-5.49) p 0.000
Current smoker	4.16 (2.24-7.75) p 0.000
Living arrangement	
Living with family	1
Not living with family	1.30 (0.97-1.74) p 0.083
Years of clinical experience	
< 1	1
> 10	1.55 (0.79-3.03) p 0.200
Hospital sector	
Governmental	1
Private	1.70 (1.07-2.70) p 0.025
Working shift	
Day	1
Night	1.46 (0.98-2.18) p 0.065

CI, confidence interval; AOR, odds ratio; * Adjusted odds ratio for age and gender.

### 3.4 Associated sociodemographic factors of anxiety among RTs’ staff

Regarding anxiety, the binary logistic regression analysis revealed that RTs who were (divorced, widowed or separated) were almost five times more likely to experience anxiety compared to single RTs [OR: 4.61 (95% CI: 1.42 to 14.92); p = 0.011]. RTs who did not live with their family were almost six times more likely to experience anxiety compared to those who lived with their family [OR: 5.70 (95% CI: 1.32 to 24.44); p = 0.019]. Former smokers and current smokers were three times and six times more likely to experience anxiety compared to non-smokers RT [OR: 3.30 (95% CI: 1.57 to 6.96); p = 0.002] and [OR: 6.33 (95% CI: 2.29 to 17.50); p < 0.001]. RTs who worked in private hospitals were two times more likely to experience anxiety compared to those who worked in government hospitals [OR: 2.40 (95% CI: 1.22 to 4.68); p = 0.011]. Those who worked night shift were two times more likely to experience anxiety compared to those who worked day shift [OR: 2.47 (95% CI: 1.38 to 4.42); p = 0.002]. **Table 3** summarizes all the predictors of anxiety.

Multivariate regression analysis identified that RTs who were (divorced, widowed or separated) were four times more likely to experience anxiety compared to single RTs [AOR: 4.46 (95% CI: 1.34 to 14.94); p = 0.015]. RTs who did not live with their family were five times more likely to experience anxiety compared to those who lived with their family [AOR: 5.48 (95% CI: 1.28 to 23.53); p = 0.022]. Former smokers and current smokers were three times and six times more likely to experience anxiety compared to non-smokers RT [AOR: 3.36 (95% CI: 1.58 to 7.15); p = 0.002] and [AOR: 6.65 (95% CI: 2.39 to 18.48); p = 0.000]. RTs who worked in private hospitals were two times more likely to experience anxiety compared to those who worked in government hospitals [AOR: 2.32 (95% CI: 1.18 to 4.55); p = 0.015]. Those who worked night shift were two times more likely to experience anxiety compared to those who worked day shift [AOR: 2.31 (95% CI: 1.28 to 4.15); p = 0.005]. **Table 4** summarizes all the predictors of anxiety.

### 3.5 Associated sociodemographic factors of depression among RTs’ staff

Regarding depression, the binary logistic regression analysis revealed that female RTs were one time more likely to experience depression compared to RT males [OR: 1.47 (95% CI: 1.03 to 2.10); p= 0.035]. RTs who were (divorced, widowed or separated) were almost seven times more likely to experience depression compared to single RTs [OR: 6.54 (95% CI: 2.35 to 18.15); p < 0.001]. Former smokers and current smokers were three times and almost four times more likely to experience depression compared to non-smokers RT [OR: 3.10 (95% CI: 1.78 to 5.30); p < 0.001] and [OR: 3.91 (95% CI: 2.12 to 7.24); p < 0.001]. RTs who did not live with their family were almost two times more likely to experience depression compared to those who lived with their family [OR: 2.28 (95% CI: 1.06 to 4.86); p = 0.033]. RTs with more than 10 years of clinical experience were almost three times more likely to experience depression compared to those with less than a year of experience [OR: 2.78 (95% CI: 1.41 to 5.45); p = 0.003]. RTs who worked in private hospitals were almost two times more likely to experience depression compared to those who worked in government hospitals [OR: 1.76 (95% CI: 1.11 to 2.79); p = 0.016]. RTs who worked night shift were one time more likely to experience depression compared to those who worked day shift [OR: 1.58 (95% CI: 1.06 to 2.35); p = 0.023]. **Table 3** summarizes all the predictors of depression.

Multivariate regression analysis identified that RTs who were (divorced, widowed or separated) were almost six times more likely to experience depression compared to single RTs [AOR: 5.91 (95% CI: 2.09 to 16.70); p = 0.000]. Former smokers and current smokers were three times and four times more likely to experience depression compared to non-smokers RT [AOR: 3.15 (95% CI: 1.81 to 5.49); p = 0.000] and [AOR: 4.16 (95% CI: 2.24 to 7.75); p = 0.000]. RTs who worked in private hospitals were almost two times more likely to experience depression compared to those who worked in government hospitals [AOR: 1.70 (95% CI: 1.07 to 2.70); p = 0.025]. **Table 4** summarizes all the predictors of depression.

## 4 Discussion

To the best of our knowledge, only one study has investigated the incidence rate of depression, anxiety, and stress among RTs in Saudi Arabia since the COVID-19 pandemic (16). Consequently, this study aimed to assess the prevalence of depression, anxiety, and stress in addition to identifying their associated sociodemographic factors among RTs in Saudi Arabia after COVID-19. Our study outcomes revealed that stress, depression and anxiety were prevalent psychological conditions among RTs as the majority experienced moderate levels of depression and anxiety, although they had normal stress levels. Additionally, RTs who were female, divorced, widowed or separated, former or current smokers, night shift workers, private hospital employees, aged 41 or older, had more than 10 years of clinical experience, and who were not living with their families were more suspectable to anxiety, depression and stress.

Our study findings showed that the prevalence rates of depression, anxiety, and stress among RTs were 81.3%, 89.6%, and 45.5%, respectively, although most of the study participants reported moderate levels of depression and anxiety and normal levels of stress. These results are comparable to numerous related studies conducted among healthcare professionals globally. According to a recently published study of 218 RTs in Canada, over half of the participants reported experiencing symptoms of depression (52%), anxiety (51%), and stress (54%) (12). In contrast, the prevalence of anxiety was comparatively low in a cross-sectional study conducted on a small sample of 34 RTs in Saudi Arabia, where it was found that only 20.6% of the participants had mild to moderate levels of anxiety (16). Among physicians, Appiani et al. reported that 22%, 44%, and 94% of 440 medical doctors in Argentina screened positively for depression, anxiety, and stress symptoms, respectively (34). In a relevant study carried out by Dave et al. on 520 resident doctors in India, it was discovered that 27.71% of them were depressed, 36.58%experienced anxiety, and 24.24% were stressed (35). Furthermore, a related study on 994 Brazilian psychologists revealed that 30-40% of them had experienced moderate levels of depression, while 25-30% of them experienced moderate levels of anxiety and stress (36). Similar findings were found in several related studies among physicians in Saudi Arabia, Pakistan, Bangladesh, Malaysia, and Brazil (37–41). Among dentists, an exploratory cross-sectional study of 998 Brazilian dentists showed that depression, anxiety, and stress were prevalent among 46-47% of the study participants (42). Additionally, analogous findings revealed that 36-46% of dental staff in China and India had depression and anxiety (43, 44). Likewise, a pertinent study conducted among 238 pharmacists in Malaysia showed that the prevalence rates of depression, anxiety, and stress were 28.2%, 40.8%, and 17.6%, respectively (23). As such, a relevant study carried out by Zhang et al. on a sizable sample of pharmacists in China indicated that 41.9% and 29.4% of them reported mild to severe symptoms of anxiety and depression, respectively (45). Among nurses, a related study conducted by Bhandari et al. on 301 nursing staff in Nepal showed that 85.72% of them were depressed, 62.80% experienced anxiety, and 49.84% stress (46). Moreover, Kaushik et al. demonstrated that the incidence rates of depression, anxiety, and stress among Indian nurses were 71%, 74%, and 51%, respectively (24). Other studies have reported identical findings regarding nursing staff in Iran and Brazil (47, 48). In a study carried out by Nadeem et al. on 189 physiotherapists in Pakistan, 26.4%, 30.2%, and 36.5% of the study participants reported having moderate to severe symptoms of depression, anxiety, and stress, respectively (49). In addition, a recent study of 150 medical imaging technologists in Pakistan showed that 35.3% of them were abnormally depressed and 10.7% of them had anxiety (50). The variances in the reported studies could be explained by differences in sample size or measurement tools.

Several associated factors are significantly associated with higher scores on the DASS-21 tool. For instance, our study outcomes corroborated that female RTs were one time more likely to experience depression and stress compared to male RTs. These findings correspond with a related study that involved 601 healthcare professionals in Vietnam, which found that women had a higher likelihood of experiencing psychological conditions, with the prevalence rates of depression and stress among women being 50% and 38%, respectively, compared to 39% and 34% for men (26). Similarly, Hassan et al. indicated that female physicians who had previously contracted COVID-19 during the pandemic had higher odds of suffering from depressive symptoms (39). In addition, a related study on Saudi Arabian physiotherapists revealed that women were more vulnerable to depressive episodes than their male counterparts (25). As such, similar research has shown that the female gender was a potential factor concerning the possibility of stress (51). However, several related studies have revealed that female healthcare practitioners were more likely to report symptoms of anxiety (22, 38, 40, 45).

Additionally, it has been found that former and current smokers were one to three times and two to six times, respectively, more likely to experience depression, anxiety, and stress compared to non-smoking RTs. Analogous findings were found in a study of 715 nurses conducted in Saudi Arabia, which revealed that smoking was substantially associated with a higher prevalence of psychological disorders and an increased association of experiencing symptoms of depression and anxiety (27). Similarly, a related study reported that the smoking habit was proven to be a significant predictor that raises the likelihood of both depression and anxiety (52). Furthermore, a cross-sectional study by Fond et al. revealed that tobacco smoking was an associated behavior that was intimately linked to an increased incidence of depression among healthcare workers (53). Based on these outcomes, additional studies are needed to investigate the association between smoking status and stress among RTs in Saudi Arabia.

In terms of marital status, our study findings showed that RT staff who were divorced, widowed, or separated were three to seven times more likely to experience depression, anxiety, and stress compared to single RTs. These findings may be attributable to the social support that families offer, which enhances the well-being of married people. In this context, a pertinent study concluded that nurses who were divorced or widowed had a considerably increased association with experiencing depressive and anxiety symptoms in comparison with those who were married (27). Nayak et al. also pointed out that single healthcare practitioners had a significantly higher incidence of depression, anxiety, and stress relative to their married colleagues (22). According to Godifay et al., healthcare professionals who were divorced, widowed, or separated were more likely to experience work-related stress (28). A related study demonstrated that divorced individuals were significantly more inclined to exhibit anxiety and depressive symptoms (25). Additionally, Kakemam et al. found a statistically significant association between marital status and depression, whereby married nurses were less likely to experience depressive episodes compared to their unmarried peers (51). Prior corroborating research has demonstrated that married healthcare providers were effectively protected against the onset of depression symptoms (40, 44). Furthermore, it has been discovered that RTs who do not live with their families are two to six times more likely to experience depression, anxiety, and stress compared to those who live with their families. Our study outcomes emphasize the benefits of family living for a healthcare professional’s psychological and social stability. These results were in line with a cross-sectional study of Saudi Arabian physiotherapists by Abdulghani et al. which revealed that healthcare workers who did not live with their families had greater rates of depression and anxiety (25). Conflicting findings were found in a relevant study of healthcare workers in Saudi Arabia during the COVID-19 outbreak, indicating that residing with family members was predicted to increase depression and anxiety (9). Meanwhile, Alzaid et al. observed that living with family members was significantly correlated with an increase in anxiety symptoms (54). Additionally, earlier studies revealed that living alongside a child or an elderly person substantially increased the likelihood of developing psychological conditions (17, 55, 56).

Concerning age and work experience, our study results showed that RTs with more than 10 years of clinical experience were almost two to three times more likely to experience stress and depression compared to those with less than a year of clinical experience. Also, RTs who were 41 years old and older were almost two times more likely to experience stress compared to those who were 20 to 30 years old. Similarly, Pei et al. found that pharmacy staff aged 30 and over had twice the odds of experiencing stress than younger staff. Also, they discovered that employees with more than three years of practical experience had a greater association with being depressed and were less inclined to suffer from stress compared to their inexperienced counterparts (23). According to a systematic review by Spoorthy et al., older healthcare providers reported high levels of stress during the COVID-19 crisis as a result of physical exhaustion and long working hours (57). Additionally, a similar study demonstrated that healthcare professionals with more than five years of clinical experience had considerably greater levels of work-related stress than junior employees (28). On the other hand, a cross-sectional study of nursing staff in Iran showed that nurses over the age of 40 years old encountered less stress compared to younger staff, while nurses with fewer than 10 years of work experience reported higher rates of stress than experienced nurses (47). Another study pointed out that younger healthcare practitioners with less than ten years of experience exhibited greater levels of stress and depression compared to their more experienced and older counterparts (22). Several relevant studies did not agree with our study findings, as they showed that the younger and junior staff were more susceptible to depression and stress than the older and senior staff (58–60).

Interestingly, the study findings demonstrated that RT staff who work in private hospitals were one to two times more likely to experience depression, anxiety and stress compared to RTs who worked in government hospitals. These findings were relatively consistent with a recent study that discovered statistically significant differences between the working sector and mental health symptoms, with nursing staff employed in the private sector being more depressed, anxious and stressed due to poor job satisfaction than those working in the government sector (24). In contrast, a recent study conducted in Saudi Arabia showed that dentists working in public facilities were moderately depressed and severely anxious (52). Numerous studies have indicated that employment in the private sector was significantly associated with a lower likelihood of experiencing severe depressive and anxiety symptoms (61, 62).

In addition, it was reported in our study that RTs who worked the night shift were one to two times more likely to experience depression and anxiety compared to those who worked the day shift. Likewise, Li et al. demonstrated that working on the night shift adversely influenced the psychological well-being of the nursing staff by raising the incidence rate of anxiety and depression (29). Furthermore, Peng et al. indicated that night and rotating shifts were highly correlated with a greater probability of developing depression and anxiety (63). According to a recently published systematic review, nursing staff who worked night shifts were more likely to have depressive symptoms than their morning shift counterparts (64). Therefore, reducing workload and psychological stress, and offering a flexible duty schedule may contribute positively to alleviating symptoms of depression and anxiety among RTS, which improves the quality of health care and decreases medical errors.

## 5 Strength and limitations

Our current study is unique since there is a dearth of similar research that investigates the prevalence of depression, anxiety, and stress among RTs in Saudi Arabia. Additionally, this study involved a widely representative sample size of the target population from a variety of regions in both the private and public sectors to generalize the results across the country. Nevertheless, our study may be somewhat limited due to the use of a cross-sectional design, convenience sampling techniques, and the self-reported survey which provided the subjective data and which may have led to partial recall bias. Additionally, this study was conducted at a highly stressful period in human history, COVID-19 pandemic, which may alter our findings. Moreover, our sample was predominantly male and dayshift workers. Further qualitative studies are needed to explore depression, anxiety, and stress prevalence and symptoms during non-COVID-19 pandemic among RTs workers in Saudi Arabia and compare it with other healthcare providers (nurses, physicians, and physiotherapists), and examine the causes of depression, anxiety, and stress to establish preventive measures and coping strategies to improve their psychological status.

## 6 Conclusion

Depression, anxiety, and stress are common among RTs in Saudi Arabia, with a majority experiencing moderate levels of depression and anxiety, requiring attention and further intervention to mitigate the effect of these psychological conditions. Several associated sociodemographic factors such as being female, divorced, widowed, or separated, being a former or current smoker, being a night shift worker, being a private hospital employee, being aged 41 or older, having had more than 10 years of clinical experience, and not living with one’s family may contribute to the development of psychological conditions.

## Data availability statement

The raw data supporting the conclusions of this article will be made available by the authors, without undue reservation.

## Ethics statement

The studies involving humans were approved by Institutional Review Board at King Abdulaziz University with reference number (EC2023-026). The studies were conducted in accordance with the local legislation and institutional requirements. The participants provided their written informed consent to participate in this study.

## Author contributions

AA: Conceptualization, Data curation, Formal analysis, Funding acquisition, Investigation, Methodology, Project administration, Resources, Software, Supervision, Validation, Visualization, Writing – original draft, Writing – review & editing.
